# Improved methylene blue adsorption from an aqueous medium by ozone-triethylenetetramine modification of sawdust-based biochar

**DOI:** 10.1038/s41598-023-39495-7

**Published:** 2023-08-01

**Authors:** Mohamed A. Hassaan, Murat Yılmaz, Mohamed Helal, Mohamed. A. El-Nemr, Safaa Ragab, Ahmed El Nemr

**Affiliations:** 1 National Institute of Oceanography and Fisheries (NIOF), Kayet Bey, Elanfoushy, Alexandria, Egypt; 2 Department of Chemical Engineering, Faculty of Engineering, Osmaniye Korkut Ata University, 80000 Osmaniye, Turkey; 3 Department of Chemical Engineering, Faculty of Engineering, Minia University, Minia, 61519 Egypt

**Keywords:** Environmental sciences, Environmental chemistry, Chemical engineering

## Abstract

In this study, sawdust biochar-O_3_-TETA (SDBT), a novel biochar, was prepared via treatment with 80% sulfuric acid, followed by oxidation by ozone and subsequent treatment with boiling Triethylenetetramine (TETA). Characterization studies of the prepared SDBT adsorbent were performed with SEM–EDX, BET, XRD, BJH, FT-IR, DTA and TGA analyses. The adsorption efficiency of MB dye by SDBT biochar from water was investigated. Methylene Blue (MB) dye absorption was most effective when the solution pH was 12. The maximum removal % of MB dye was 99.75% using 20 mg/L as starting MB dye concentration and 2.0 g/L SDBT dose. The *Q*_m_ of the SDBT was 568.16 mg/g. Actual results were fitted to Temkin (TIM), Freundlich (FIM), and Langmuir (LIM) isotherm models. The experimental results for SDBT fitted well with all three models. Error function equations were used to test the results obtained from these isotherm models, which showed that the experimental results fit better with TIM and FIM. Kinetic data were investigated, and the pseudo-second-order (PSOM) had *R*^2^ > 0.99 and was mainly responsible for guiding the absorption rate. The removal mechanism of the MB dye ions in a base medium (pH 12) may be achieved via physical interaction due to electrostatic interaction between the SDBT surface and the positive charge of the MB dye. The results show that SDBT effectively removes the MB dye from the aqueous environment and can be used continually without losing its absorption efficiency.

## Introduction

Our world is evolving into new perspectives with the growing population and technological advances. In the current period, water consumption has dramatically increased. Conserving water resources to ensure future water security is more critical now than ever. Households, industry, and agriculture produce large amounts of sewage containing various pollutants. Chemical compounds that place a heavy burden on the ecosystem can be listed as heavy metals^1–5^, drugs^6,7^, pesticides^8–10^, hydrocarbons^11,12^ and dyes^13–17^. Dyes are one of the most critical categories of contamination^18^. Synthetic dyes are the most commonly used dye type in textile, leather, and many other industries^19^. Because these dyes are toxic, non-biodegradable and carcinogenic, they provide a grave risk to the environment and public health^20,21^. The average amount of unprocessed dyes released into water bodies is around (0.7–2.0) × 10^5^ tons per year^22^. Azo dyes are used too much because they have a wide variety of colors and are the most compatible among all synthetic dyes, creating cancer-causing substances^18^.

The main methods of treating effluents from dyes factories in the industry can be listed as electrochemical treatment^23^, chemical oxidation^24^, biological treatment^25^, photo-degradation^26–29^, coagulation/flocculation^30^, advanced oxidations^31–34^ and adsorption treatment^15–17,19,35^. However, most methods have disadvantages, such as being able to partially remove stubborn and non-biodegradable dyes, being uneconomical, and creating undesirable by-products. However, among the methods used in dye effluent water treatment, adsorption is much more advantageous than other methods owing to its simplicity of design, affordability, and ease of usage^36^. However, scientists continue their studies to develop both effective and cheaper adsorbent materials since the production and processing of activated carbon, which is the greatest used adsorption method, is an expensive process^3,37–39^. In this way, biochar obtained from waste products and large mass also prevents the waste of resources. In the literature, biochars are obtained by gasification or pyrolysis of various biomass in an inert gas environment, such as argon or nitrogen, at temperatures higher than 350 °C^40^. Biochars have more functional groups despite having a lesser surface area and pore capacity than activated carbons^41,42^.

The efficiency of biochars in wastewater removal is directly associated with the number and variety of functional groups that will be included in its structure. This can be attained by making chemical changes to the biochar surface. Carbon surface activation, oxidation, nanoscale formation, and metal impregnation are among the methods used to increase the biochar absorption capacity^43^. The biochar absorption capacity can be increased by attaching amino groups to the surface^41^. It is feasible to increase the functional group number by treating biochars with different acids (HNO_3_, H_3_PO_4_ or H_2_SO_4_), different bases (H_2_SO_4_, NaClO, HNO_3_, KOH or H_3_PO_4_) or oxidizing reagents (KMnO_4_, NaClO, NH_3_H_2_O, H_2_O_2_ or (NH_4_)_2_ S_2_O_8_))^42,44,45^. It has been observed in many studies that biochars are loaded with nanometals, improving oxidation resistance, increasing the surface area, expanding the adsorption sites and having high thermal stability^46^. Agents such as FeSO_4_, H_2_, NH_3_H_2_O, Na_2_SO_3_ and aniline are among the most commonly used reducing agents^47^. There have been many studies on the removal of many pollutants with activated carbons derived from agricultural biomass. Coconut husk^48^, sesame hull^49^, olive stone^50^, green algae *Ulva lactuca*^51^, orange peel^52^, tea waste^53^, gulmohar^54^, rice straw^55^, potato^56^, Macore fruit^57^, Barely straw^58^, red algae *Pterocladia capillacea*^59^, mandarin peel^60^, sugarcane bagasse^52^, coffee bean husks^61^, watermelon peel^62^ and peanut husk^63^ are some of this agricultural biomass.

Numerous elements, including its high carbon content, aromatic functional groups containing oxygen, and high porosity, may have an impact on its structure. Its surface area, stable molecular structure, and porosity encourage the adsorption of contaminants on it^3^. According to Fuertes et al.^1^, in the first 15 min at starting pH 2, direct red 23 and 80 were shown to have adsorption capacities of 10.72 and 21.05 mg/g for biochar made from maize Stover. On the other hand, Wang et al.'s^14^ investigation focused on the adsorption of harmful metals onto hickory wood-derived biochar that had been KMnO_4_-treated. According to their findings, the biochar they produced displayed adsorption capacity 153.1 mg/g of Pb, 34.2 mg/g of Cu, and 28.1 mg/g of Cd. This variation in adsorption might be brought about by these metals' varying valences and their affinity for the biochar. Additionally, Sun et al.^16^ observed that increasing the amount of biochar (made from swine manure) from 1 to 8 g/L led to a greater number of active sites that could be used to adsorb methylene blue. When methyl violet initial concentrations ranged from 40 to 816 mg/L, Xu et al.^18^ used peanut straw biochar and achieved an adsorption capacity of 104.61 mg/g.

Many studies deal with the removal of MB dye through adsorption, but in the author knowledge, the modification of sulphonated SDB biochar by treating SDB with Ozone followed by modification with TETA is used for the first time for MB removal. Therefore, this work evaluated the effectiveness of a novel sawdust biochar-O_3_-TETA (SDBT) adsorbent for the adsorption of MB dye from an aqueous solution. This adsorbent is readily available, biodegradable, non-hazardous, and affordable. The impacts of SDBT dosage, pH, beginning adsorbent concentration, and contact duration between SDBT and MB dye were tested as removal conditions for MB dye from an aqueous solution. To study the adsorption structure and maximum adsorption capability of SDBT adsorbent adsorption isotherms were also tested.

## Materials and methods

### Resources and instrument

In order to make biochar, wood sawdust from a nearby wood market in Alexandria, Egypt, was gathered and utilized as a raw material. Methylene blue dye (Assay 99%) was used to create the standard stock solution; it was purchased from Sigma Aldrich in the USA. This investigation utilized Pg Instrument model T80 UV/Visible High-Performance Double Beam Spectrophotometer, A JS Shaker (JSOS-500), and pH meter JENCO (6173) were utilized. Based on a thermodynamic model, the adsorption–desorption isotherm of SDB, SDBO, and SDBT biochars was conducted in N_2_ environment. N_2_ adsorption at 77 K was used to determine the surface area (SA) of the SDB, SDBO, and SDBT biochars using a surface area and pore analyzer (BELSORP—Mini II, BEL Japan, Inc.)^64,65^. Surface area (*S*_BET_) (m^2^/g), monolayer volume (*V*_*m*_) (cm^3^ (STP), average pore diameter (MPD) (nm), total pore volume (*p*/*p*_0_) (cm^3^/g) and energy constant (*C*) values of SDB, SDBO, and SDBT biochars were calculated using this plot. The mesoporous SA (*S*_*mes*_), microporous SA (*S*_*mi*_), the volume of mesoporous (*V*_*mes*_), and the volume of microporous (*V*_*mi*_) of SDB, SDBO, and SDBT biochars were assessed using the Barrett–Joyner–Halenda (BJH) model. The computations were carried by with the BELSORP analysis program software. The BJH method^66^ was also used to extract the pore size distribution from the desorption isotherm. The form of the biochar's surface was examined using a scanning electron microscope (SEM) (QUANTA 250). The functional groups on the surface of biochars were investigated using Fourier Transform Infrared (FTIR) spectroscopy (VERTEX70) coupled with an ATR unit model V-100. On the surface of the SDB, SDBO, and SDBT biochars, IR-observable functional groups were found in the 400–4000 cm^–1^ wavenumber range with resolution of 0.5 cm^–1^. The SDT650-Simultaneous Thermal Analyzer instrument was used for thermal analyses with a temperature range of 50–1000 °C and a ramping temperature of 5 °C/min. X-ray diffractograms (XRD) was investigated using a Bruker Meas Srv (D2 PHASER) (D2-208219)/D2-2082019 diffractometer working at 30 kV and 10 mA with a 2*θ* range of 5–80 and a Cu tube (*λ* = 1.54).

### Methods

#### Sawdust biochar (SDB) preparation

To remove dust, the collected wood sawdust was thoroughly washed with tap water multiple times. It was then dried for 24 h at 105 °C in an oven. The samples were first cooked in a refluxed system at 280 °C for 4 h with 100 g of sawdust in a 400 mL solution of 80% H_2_SO_4_^2,3,41,42,44^, after which, the samples were filtered, washed with distilled water (DW) until the washing solution became neutral, and then washed with EtOH. The weight of the finished biochar product (45 g) was ascertained after oven drying at 105 °C. This process produced biochar which was named SDB.

#### Preparation of ozonized saw dust biochar (SDBO)

Following preparation, the SDB was treated with ozone in DW. For ozone treatment of SDB, 200 mL of DW was used to ozonate 40 g of produced SDB for 2 h. After filtering, the SDB was then washed with ethanol and DW^2,3^. The ozonated SDB was oven-dried overnight at 105 °C and branded as SDBO.

#### Treatment of SDBO with TETA

TETA (100 mL) was used to boil 30 g of SDBO biochar for 4 h, after which it was cooled, filtered, and washed with DW and EtOH. The solid biochar was branded as SDBT after drying for 24 h at 105 °C.

### Absorption measurement for methylene blue dye

By dissolving 1.0 g of MB dye in 1000 mL of DW, a stock solution of the dye (1000 mg/L) was created, and this solution was diluted to achieve the necessary concentration for the removal test and the standard curve. To assess the absorption capacity, thermodynamic, and kinetic properties of SDBT, which was created from SDB, batch adsorption experiments were used. A series of Erlenmeyer flasks (300 mL) containing 100 mL of various MB dye solution concentrations and varying doses of biochar were shaken at 200 rpm for a predetermined period. 0.1 M HCl or 0.1 M NaOH was used to change the sample pH to the required values. After separating the adsorbent from around 0.5 mL of the solution in the Erlenmeyer flask, the concentration of MB dye was measured at various intervals and in equilibrium. Spectrophotometry at λ_max_ 665 nm was used to assess the amount of MB dye present^67,68^. The absorption capacities of MB dye at equilibrium (*q*_e_) were considered from Eq. (1):

1 $$q_e=\frac{C_0-C_e}{W}\times V$$

where *q*_e_ is the MB dye amount per unit of absorbent at equilibrium (mg/g); *C*_0_ and *C*_e_ (mg/L) are the starting and equilibrium MB dye concentrations in the liquid phase, respectively; *V* is the volume of the solution (L), and *W* is the SDBT mass in gram.

#### Solution pH impact

With 100 mL of 100 mg/L starting MB dye concentration and solution pH (2–12), the impact of pH on SDBT was examined^69^.

#### The impact of the starting MB dye concentration, adsorbent dose, and contact duration

With varying starting MB dye solution concentrations (20–120 mg/L) and various dosages of SDBT biochar (0.05–4.0 g/L), the isotherm investigation for SDBT was carried out. The samples were shaken at 200 rpm, and at various time intervals at room temperature (25 ± 2 °C), the MB dye concentration was determined.

## Results and discussion

### SDB, SDBO, and SDBT characterization

#### FTIR estimation of biochar surface functional groups

**Figure 1:**
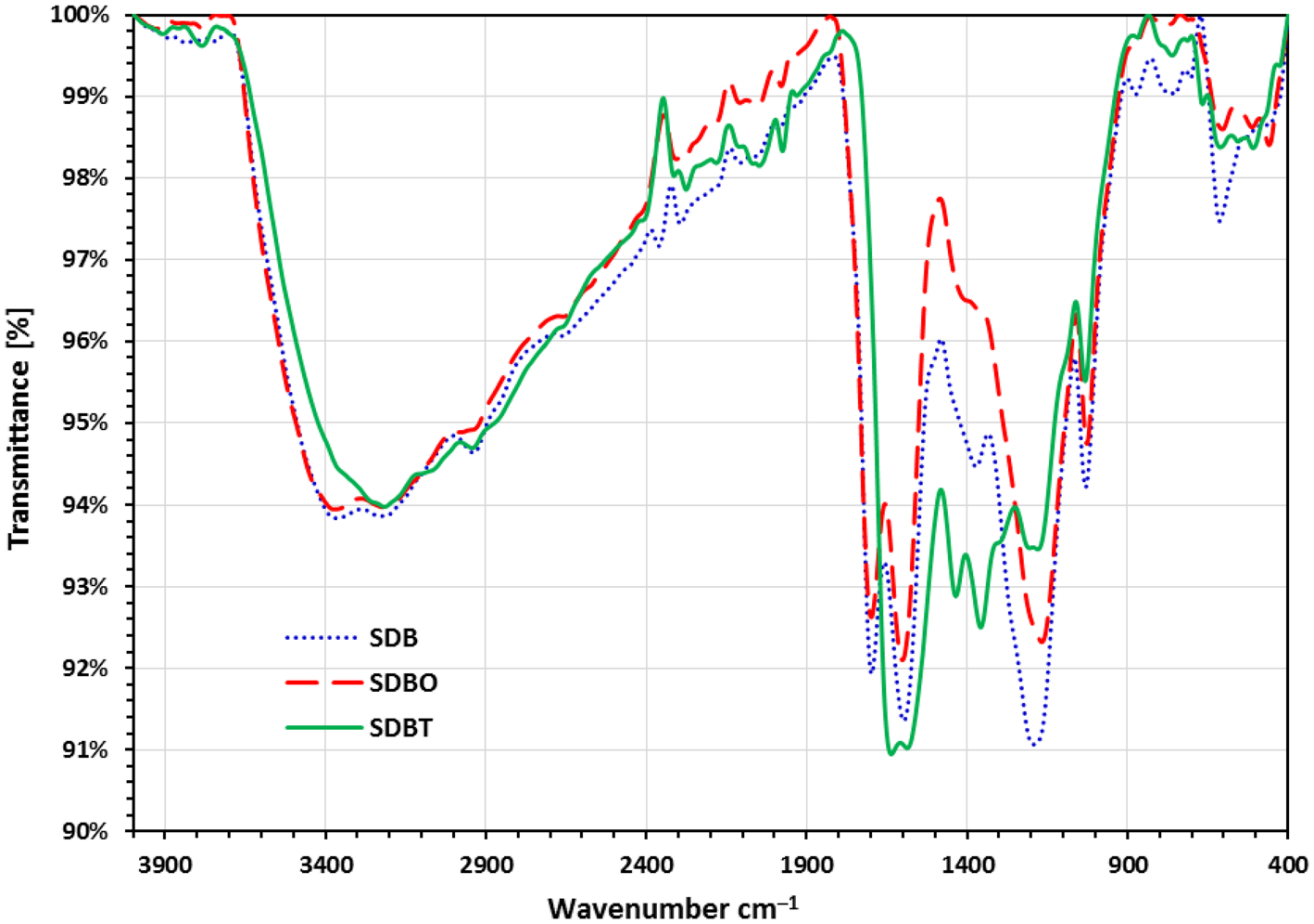
FTIR investigation of SDB, SDBO, and SDBT biochars before MB dye absorption.

SDB, SDBO, and SDBT biochars underwent FTIR analysis to identify the functional groups on their surfaces and determine the impact of alteration on the disappearance or emergence of new functional groups. SDB, SDBO, and SDBT biochars' FTIR spectra are displayed in Fig. 1. While the band at 2938.4 cm^–1^ shows the C–H stretch of the alkyl in SDB, SDBO, and SDBT biochars, the bands at 3355 and 3213 cm^–1^ represent the O–H stretching vibration that existed in these biochars. The COOH, C=C, –C–C– stretch (in-ring), and C=O are represented by the bands from 1800 to 1450 cm^–1^, which are referred to as "overtones", as band 1697.5 cm^–1^ in SDB and SDBO^70,71^. The band at 1028, 1030 and 1035 cm^−1^ in SDB, SDBO and SDBT, respectively, are correlated to the C–O–H group vibrations^72–74^.

**Figure 2:**
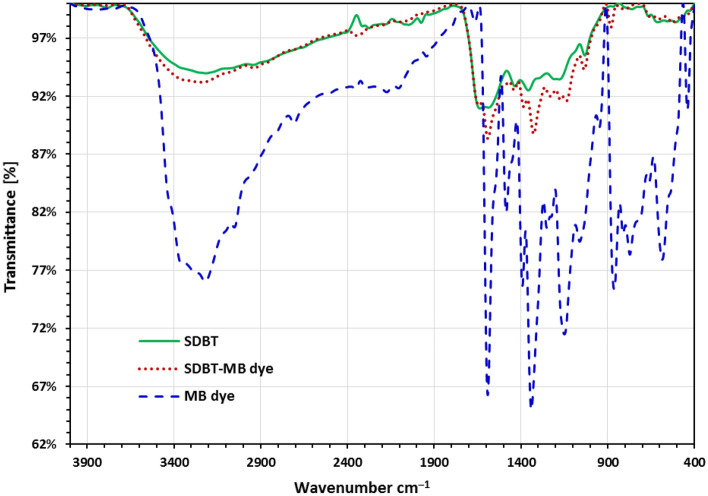
FTIR investigation of MB dye, SDBT, and SDBT biochars after contacted for 3 h with MB dye.

After being subjected to MB dye removal for three hours, Fig. 2, displays the FTIR spectrum of MB dye, SDBT, and SDBT biochars. After the biochar under test was subjected to the MB dye absorption method, it was found that all of the FTIR tests showed bands at 1616.2, 1375.1, 1323.1, 1218.9, 1147.5, 1060.7, and 902.7 cm^–1^ that are related to the MB dye. These peaks proved the adsorption of methylene blue onto SDBT biochar^51,59,71–75^.

#### SDB, SDBO, and SDBT surfaces area analysis

**Figure 3:**
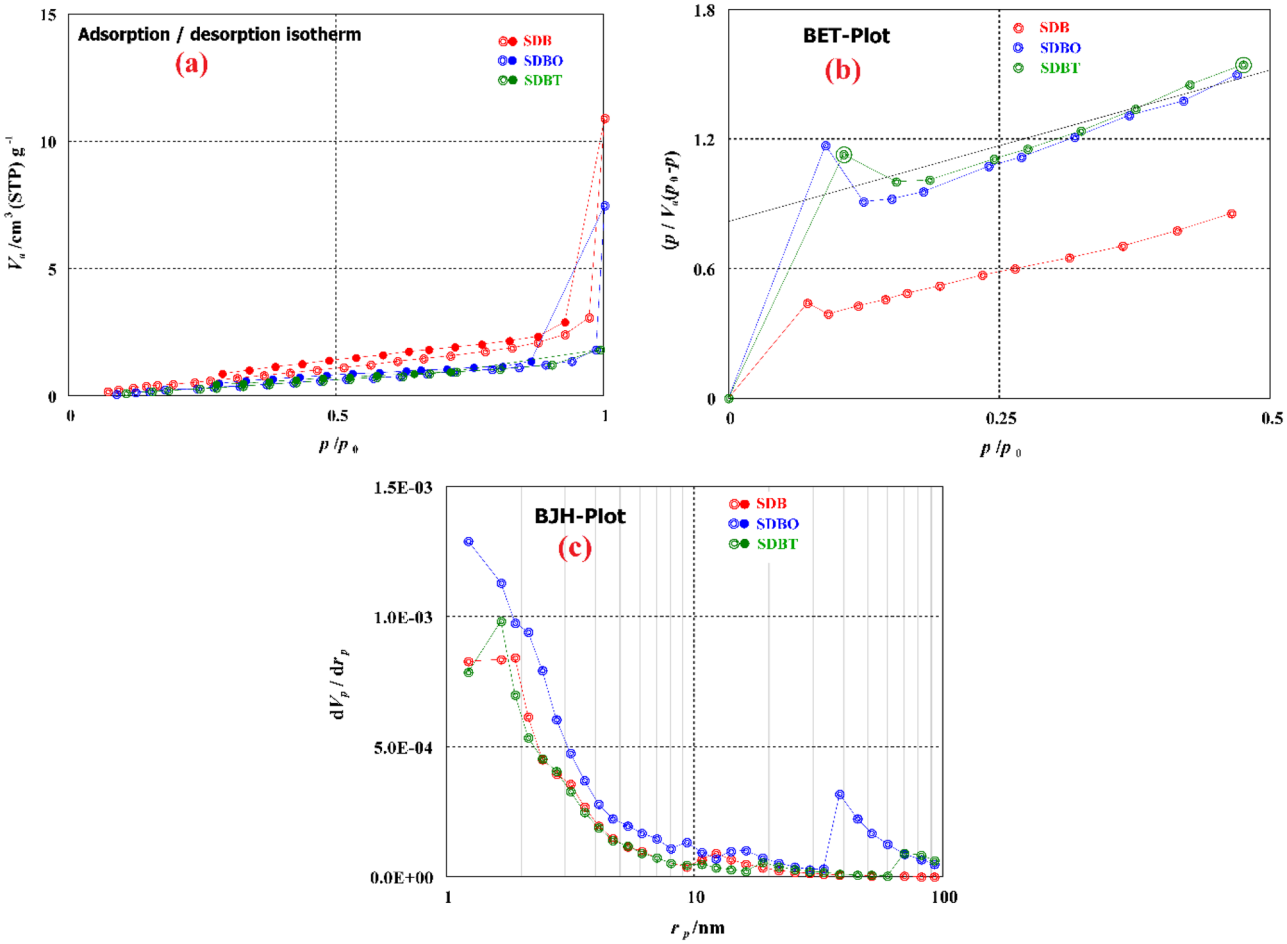
(**a**) Adsorption–desorption, (**b**) BET, (**c**) BJH investigation of SDB, SDBO and SDBT biochars.

N_2_ adsorption–desorption was used to examine how ozone and TETA treatment affected the surface characteristics of wood sawdust biochar (SDB). To determine a particular feature of biochar surfaces, BET and BJH techniques were utilized. The biochars' BET-specific surface area (SA) declined as SDB (6.61 m^2^/g) > SDBT (6.08 m^2^/g) > SDBO (1.98 m^2^/g), as seen in Fig. 3. It should be highlighted that changes have an impact on a particular surface area and that ozone modification has a more significant impact than chemical modification from TETA therapy. The average pore size shrank in the following order: SDBT (14.514 nm) > SDBO (10.716 nm) > SDB (10.07 nm), and TETA modification had a more significant impact than ozone on the reduction in pore size because of the addition of OH groups. SDB, SDBO, and SDBT biochars showed a mesoporous type and have total pore volumes of 16.664 × 10^–3^, 5.291 × 10^–3^, and 22.205 × 10^–3^ cm^3^/g. BJH results for SDB, SDBO, and SDBT biochars are shown in Fig. 3c, and their surface characteristics are included in Table 1.

**Table 1:** BJH analysis results of SDBO SDB, and SDBT biochars.

Biochar	SA (m^2^/g)	*V*p (m^3^/g)	*r*p, peak (area) (nm)
SDB	7.4265	0.018113	1.66
SDBO	2.1516	0.0057151	1.66
SDBT	6.5051	0.023449	1.66

#### Morphological surface properties of SDB, SDBO, and SDBT

**Figure 4:**
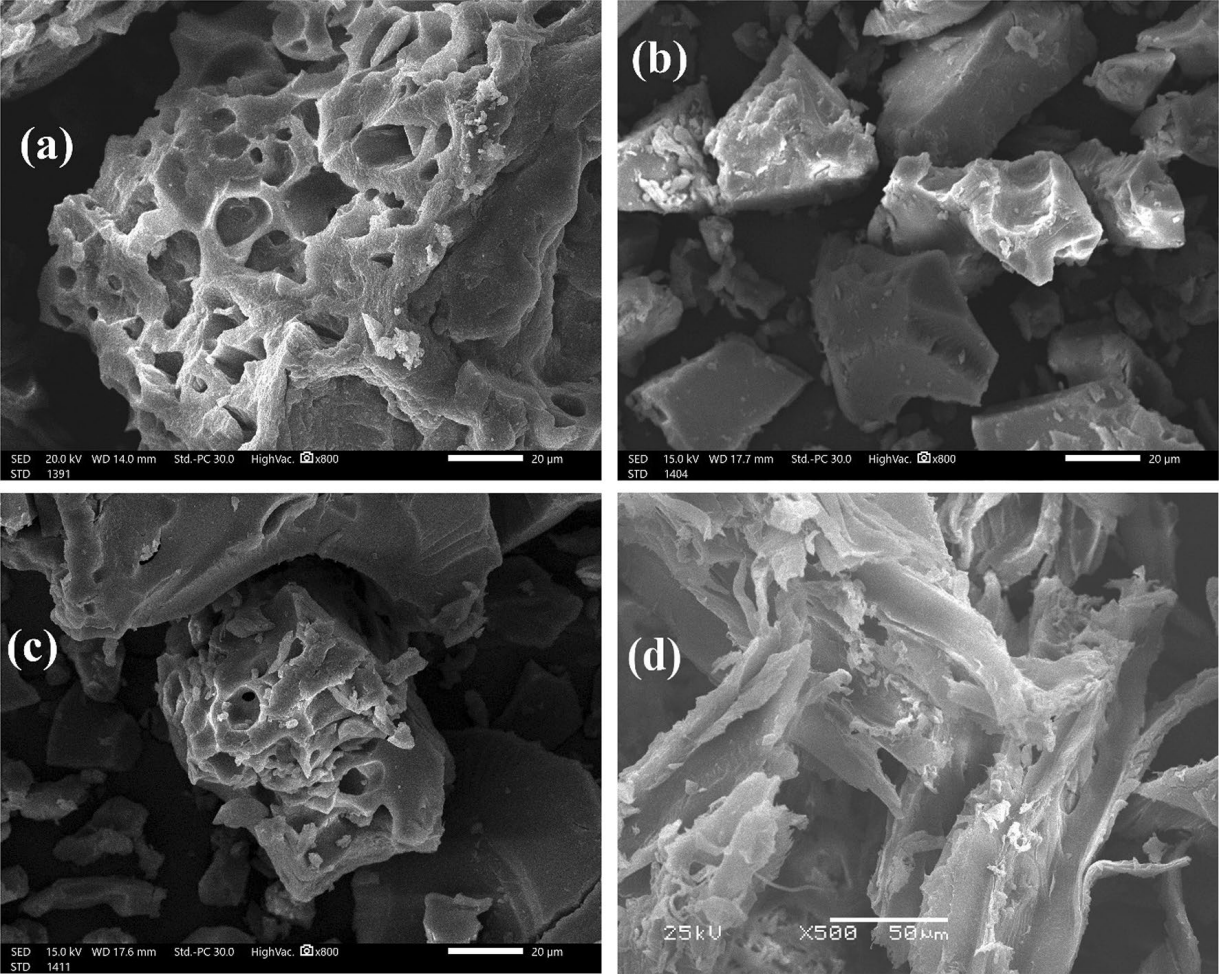
Scan electron microscope investigation of (**a**) SDB, (**b**) SDBO, (**c**) SDBT biochars, and (**d**) Raw SD material (RSD).

Figure 4 shows the results of a scan electron microscopy (SEM) analysis of the surface morphology of sawdust raw material (RSD), SDB, SDBO, and SDBT biochars. The SDB and SDBO biochars, as illustrated in Fig. 4a,b, look clean and devoid of any impurities or particulates, and no damage to the SDB's pores due to the dehydration process with 80% H_2_SO_4_ was noticed. As a result of ozone surface oxidation, Fig. 4b depicts the SDBO biochar as having a few tiny holes corresponding to the SDBO biochar's limited surface area. This validates our earlier discovery that ozone treatment of biochar in water resulted in pore blockage, which reduced surface area^72–74^. It is apparent that the ozone treatment in the water caused the pore to be blocked and the surface area of the SDBO biochar to diminish. The shape of the SDBT biochar produced by treating SDBO with TETA under boiling conditions is shown in Fig. 4c. The oxygen and sulfur groups were replaced with amino groups during this process, increasing the surface area of SDBT (6.5051 m^2^/g) over SDBO (2.1516 m^2^/g) but still falling short of SDB (7.4265 m^2^/g). No holes were seen in the RSD's SEM picture depicted in Fig. 4d.

#### SDB, SDBO, and SDBT elemental analysis

The chemical makeup of SDB, SDBO, and SDBT biochars was examined using an Energy Dispersive X-ray spectrometer (EDX). Table 2 displays the data of the analysis of the elemental percentages of SDB, SDBO, and SDBT biochars and illustrates the lack of nitrogen peak prior to TETA reagent modification. The EDX analysis of SDBT biochar revealed that 13.39% of the sample weight was nitrogen, and 1.18% was sulfur.

**Table 2:** EDX investigation results of SDB, SDBO and SDBT biochars.

Biochar	SDB		SDBO		SDBT	
Elements	Wt%	At%	Wt%	At%	Wt%	At%
Carbon	48.23	56.07	59.24	66.18	52.59	58.98
Nitrogen	NA	NA	NA	NA	13.39	12.88
Oxygen	28.15	26.98	36.90	32.04	32.84	27.65
Sulfur	23.62	16.95	3.86	2.77	1.18	0.49

*NA* not available.

#### SDB, SDBO, and SDBT thermal characterization

**Figure 5:**
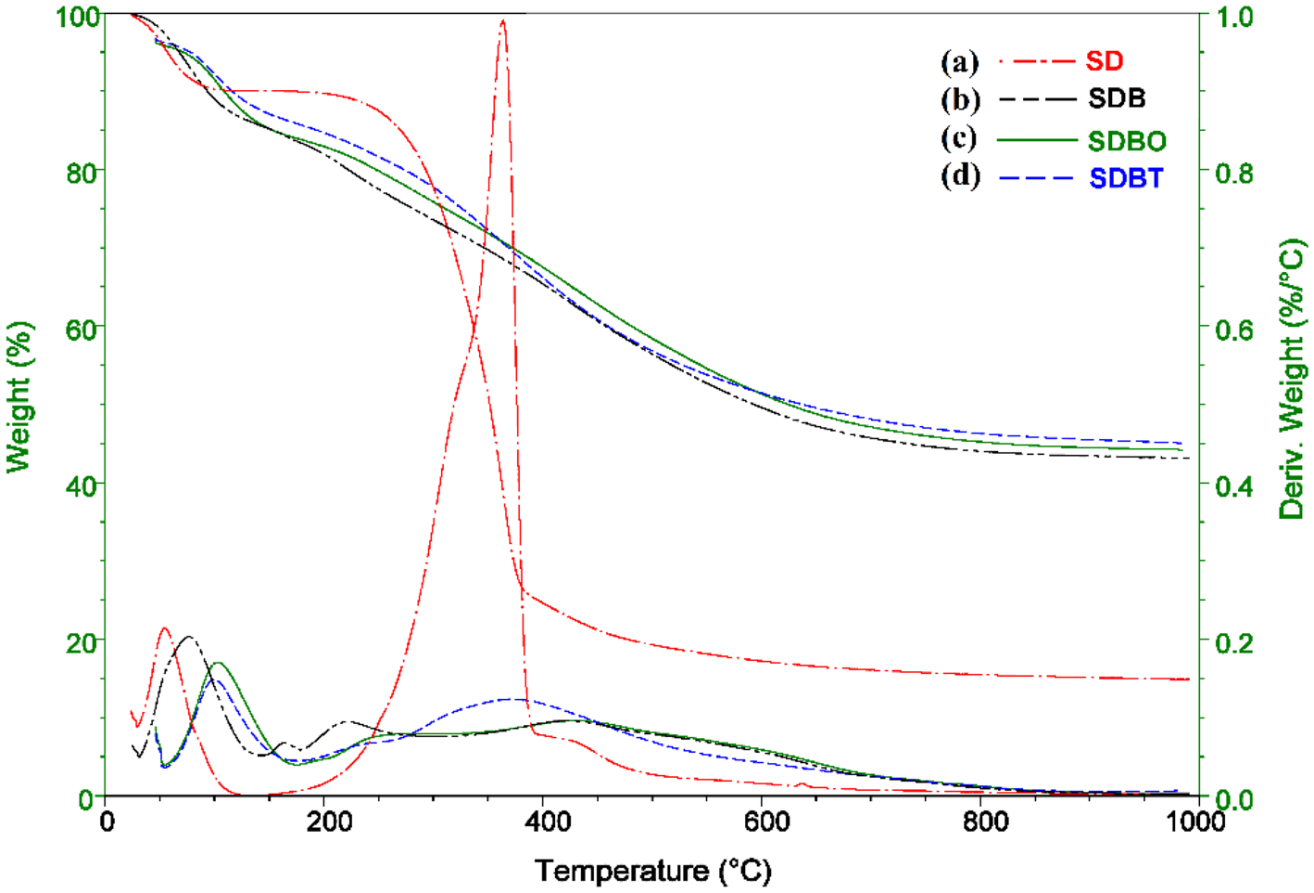
TGA investigation of (**a**) RSD material, (**b**) SDB, (**c**) SDBO, and (**d**) SDBT biochars.

Figure 5 depicts the TGA breakdown of unprocessed SD, SDB, SDBO, and SDBT biochars. The first stage of breakdown in RSD (Fig. 5a) takes place at temperatures between 50 and 140 °C and involves the loss of moisture and surface-bound water contained in the RSD, with a mass loss of around 9.84%. The second stage involves temperature between 140 and 400 °C with a considerable mass loss of around 65.41% in weight. Temperatures between 400 and 1000 °C are used in the third breakdown stage, with a weight loss of around 9.74%. About 85% of the raw sawdust sample was comprised of the three mass losses. The moisture and surface-bound water contained in the RSD sample were reflected by two significant peaks in the DTA analysis at 55.47 °C, and the substantial weight loss was indicated by a high peak at 363.62 °C (Fig. 5a). With a weight loss of around 14.30%, the first stage of breakdown takes place in the SDB biochar sample (Fig. 5b) at temperatures between 50 and 140 °C. The second phase involves temperatures between 140 and 175 °C and a weight loss of about 2.19%. Temperatures between 175 to 300 °C are used in the third breakdown stage, with a weight loss of around 9.61%. Temperatures between 300 and 1000 °C are used in the fourth breakdown process, with an estimated weight loss of 30.69% (Fig. 5b). The moisture and surface-bound water contained in the sample were represented by four significant peaks in the DTA analysis of the SDB sample at 76.22 °C, and a tiny peak representing a modest weight loss was seen at 163.09 °C (Fig. 5b). The third and fourth peaks occurred at 219.31 and 432.62 °C represented 41.21% mass loss of the SDB sample. The SDBO biochar sample analysis shows three weight loss positions with total mass loss representing 52.09% of the sample mass (Fig. 5c). At temperatures between 50 and 175 °C, the first breakdown stage takes place and results in a weight loss of around 12.26%. 8.18% of the weight was lost in the second stage at temperatures between 175 and 300 °C, while 31.65% was lost in the third decomposition process at temperatures between 300 and 1000 °C (Fig. 5c). The DTA analysis of SDBO sample represented three major peaks at 102.99 °C for moisture and surface-bound water existing in the sample and at 246.08 °C as a moderate peak represented a small weight loss and the third peak was presented at 443.57 °C showing the major mass loss percent (Fig. 5c). The analysis of SDBT biochar sample shows three weight losses positions with total mass loss represented 51.619% of the sample mass, which is almost similar to the total mass loss occurred for SDBO sample (Fig. 5d). In the first stage of mass loss, which takes place between 50 and 180 °C, there is an average weight loss of 10.86%. The second mass loss phase occurred at temperatures between 180 and 260 °C, with a modest mass loss of 4.73%. In comparison, the third mass loss step took place at temperatures between 260 and 1000 °C, with a roughly 36.02% weight loss (Fig. 5d). The DTA analysis of SDBT sample represented three distinguish peaks at 99.47 °C for moisture and surface-bound water present in the SDBT sample and at 231.49 °C as a small peak represented a 4.73% mass loss and the third peak was presented at 380.00 °C showing the major mass loss percent (Fig. 5c).

#### XRD characterization of SDB, SDBO, and SDBT

**Figure 6:**
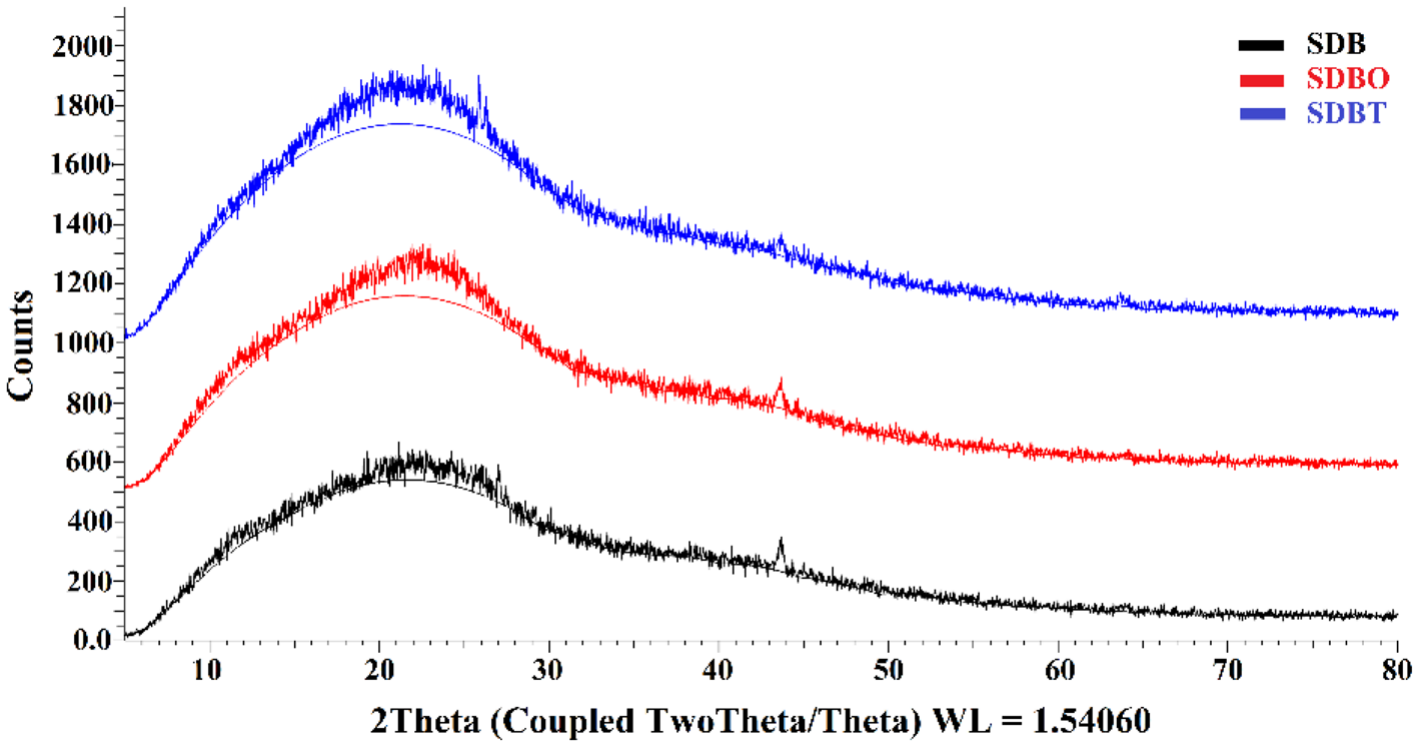
XRD analysis of prepared SDB, SDBO, and SDBT biochars.

Figure 6 displays the SDB, SDBO, and SDBT biochars' XRD. Indicating an amorphous carbon structure with aromatic sheets that are arbitrarily aligned, the wide peak in the area of 2*Ɵ* = 10–30 is indexed as C (002) diffraction peak. In contrast to SDBO, which only has one sharp peak, SDB has two peaks around 2*Ɵ* = 43.682 and 27.004, whereas SDBO has three peaks altogether. On the other hand, the structure of the SDBT biochar sample has two prominent peaks around the values of *2Ɵ* = 25.875 and 43.669, which could indicate various inorganic components mostly made of quartz and albite ^75,76^.

### Adsorption of MB dye on SDBT

**Figure 7:**
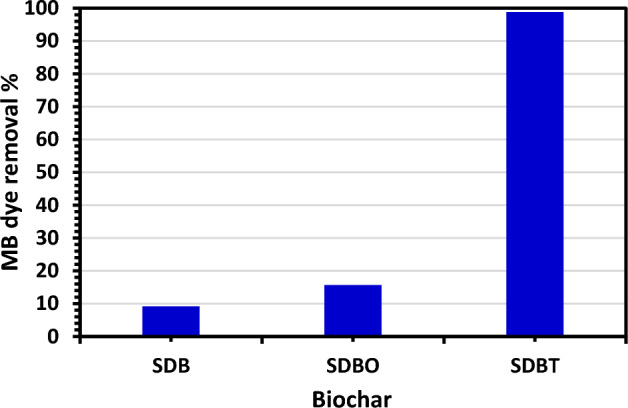
Test of MB dye removal by SDB, SDBO, and SDBT biochars (MB dye *C*_0_: 120 mg/L and 2.0 g/L biochar dose at room temperature*.*

The removal of MB dye by SDB, SDBO, and SDBT was tested to select which biochar has the highest tendency to absorb MB dye from water. Figure 7 shows the removal test of MB dye using prepared SDB, SDBO, and SDBT biochars. As seen from Fig. 7, SDB and SDBO showed a very low removal % for MB dye, while the aminated biochar SDBT showed a high ability to absorb MB dye from its water solution.

#### pH_PZC_ and ımpact of pH

**Figure 8:**
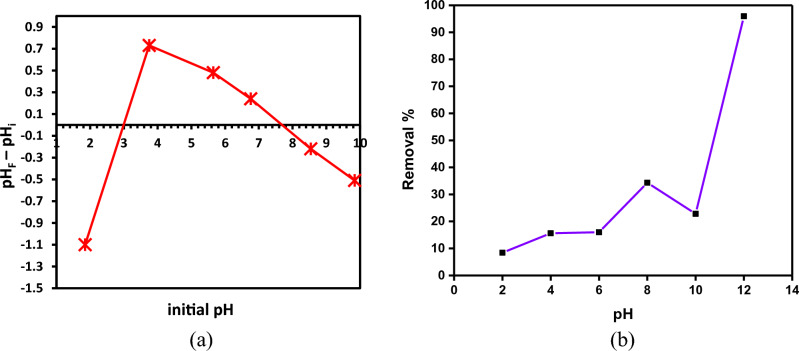
(**a**) pH_ZPC_ of SDBT (3.0 and 7.7), (**b**) Inpact of pH on the MB dye removal % by SDBT (MB = 20 mg/L, adsorbent dose = 0.5 g/L, Temperature = 25 °C).

The initial pH of the solution was plotted against the difference between the initial and equilibrium pH (Fig. 8a). The pH_PZC_ value for SDBT crossed the x-axis at two points. The pH_PZC_ values for SDBT were 3.0 and 7.7, which are in the acidic and basic ranges. To predict the adsorption process, the pH is a vital operational parameter that affects the surface charges of the SDBT as well as interfacial transport phenomena. The solution pH influences the absorption process by considerably impacting the amino, hydroxyl, and carboxyl groups on the biochar surface. The functional groups on the biochar surface can affect the occurrence of two pH_PZC_. If the concentrations of these surface functional groups are high enough and their dissociation constants are sufficiently different, biochar with multiple surface functional groups such as carboxylic acids (–COOH) and hydroxyl groups (–OH) or primary amines (–NH_2_), secondary amines (–NHR) and hydroxyl groups (–OH) can potentially display two pH_PZC_. Due to the pore structure of the biochar, the total surface charge of the material may fluctuate depending on how accessible the surface functional groups are to ions in solution. Biochar with a lot of micropores may have a more negatively charged surface as a result of more surface functional groups, which might result in a lower pH_PZC_. In contrast, biochar with a high percentage of macropores may have a surface that is less negatively charged due to the absence of as many surface functional groups, which might result in a higher pH_PZC_.

Methylene Blue (MB) dye removal on SDBT adsorbent, equilibrium studies and adsorbed quantification were performed in a 20 mg/L initial MB dye solution at a concentration of 0.5 g/L of SDBT at 25 °C. For 180 min, pH values between 2 and 12 were used to examine the MB dye's ability to bind. The pH-dependent variation of MB dye removal % is shown in Fig. 8b. Figure 8b shows that 95.96% of the MB dye is removed when the pH is 12. It can be observed that when the solution pH rose from 2 to 8, MB removal increased from 8.40 to 34.34%, dropped significantly at pH 10, and then increased to its highest level at pH 12. Jabar et al.^16^ studied the Methylene Blue dye removal using biochar made from African almond (*Terminalia catappa *L.) leaves and found that the adsorption capacity increased with increasing the solution pH from 2 to 8. This study was one of the literature studies on the removal of azo dyes. In their examination into the removal of the dye Methylene Blue using Bioadsorbent generated from Schinus molle, Razzak et al.^77^ found that raising the pH of the solution from 2 to 8 raised the adsorption percentage from 30 to 100%. The literature on Methylene Blue removal has revealed several comparable findings^78,79^. The ideal pH for SDBT's elimination of MB dye was 12.

The carboxyl (–COOH), amino (–NH_2_), and hydroxyl (–OH) functional groups on the biochar surface are extremely sensitive to the pH of the wastewater because it influences the attraction and repulsion forces that exist between the adsorbate and the adsorbent. When the pH of an aqueous solution is low, the water ionizes, depositing H_3_O^+^ on the SDBT's surface-active sites to positively charge the surface. Due to electrostatic repulsion, cationic-charged MB dye molecules cannot bind to the cationic-charged SDBT surface. So, color removal is only partially effective. The protonation of active sites on the SDBT surface relaxes as the pH of the solution rises, making it simpler for the MB dye to transfer from the aqueous solution to the surface of SDBT. The decrease in competition between H_3_O^+^ and the MB cationic dye molecules for adsorption to active sites on the adsorbent surface might account for the rise in adsorption effectiveness with rising pH. Additionally, the evolution of electrostatic interactions between cationic MB dye molecules and anionic SDBT active sites, which occurred as a result of the reappearance of –CO^–^, –OH^–^, and –NH^–^ functional groups with rising pH values, may have contributed to the rise in adsorption efficacy.

#### Contact time impact

**Figure 9:**
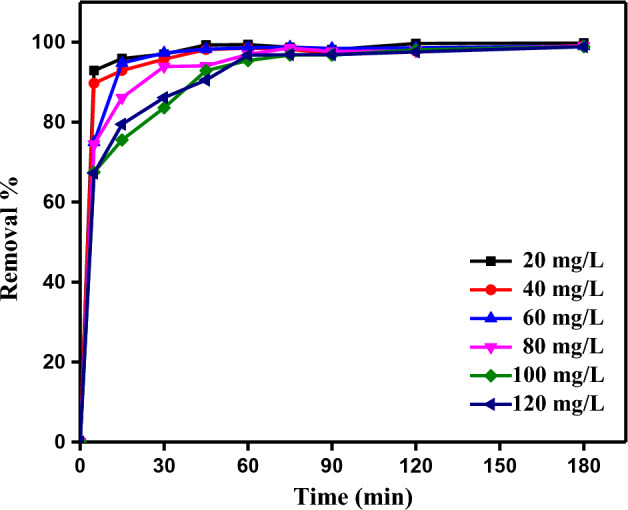
Rremoval of MB dye for 180 min using SDBT as an adsorbent (MB dye *C*_0_ = 20–120 mg/L), SDBT dose = 2 g/L, Temperature = 25 °C).

Contact time with SDBT is a significant factor in the absorption of MB dye, and for this purpose, this effect was investigated at starting MB dye concentration of 20–120 mg/L by adjusting the solution pH to 12. The MB dye adsorption to SDBT adsorbent was very fast in the first 5 min as seen in Fig. 9, and then a steady increase continued. In the first half hour, 86–97% of the total adsorption was completed. With increasing contact time, the MB dye was continuously removed. Depending on the starting MB dye concentration (20, 40, 60, 80, 100, and 120 mg/L), the elimination at 180 min was 99.75, 99.12, 99.98, 97.08, 98, 84 and 98.84%, respectively.

In cases where the solution concentration is low (20–40 mg/L), the removal efficiency is high since MB dye molecules will easily find empty active sites on the SDBT surface and adhere to them. In the opposite case, since there are too many dye molecules at high concentrations (100–120 mg/L) MB dye, each of them will not find enough empty active sites, and there will be limited removal. A comparable results were found by El Nemr et al.^62^ and Eleryan et al.^15^ when they investigated Acid Yellow 11 dye removal by different adsorbents.

#### Impact of starting MB dye concentration

**Figure 10:**
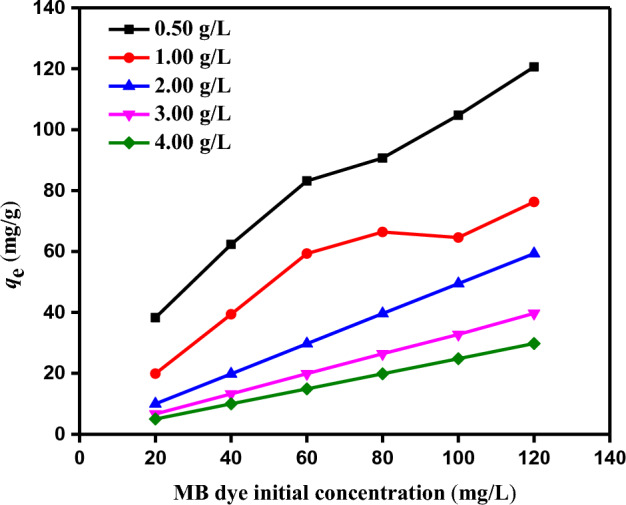
The effect of MB dye starting concentration (20–120 mg/L) using SDBT doses (0.5–4.0 g/L) on *q*_*e*_ (mg/g) at (Temperature = 25 °C).

It is possible to estimate how the initial concentration of the MB dye will impact the equilibrium adsorption capacity (*q*_e_) by using the beginning concentration of the adsorbed material, which is a crucial component of the absorption process. The initial MB dye concentration (20–120 mg/L) and adsorbent concentration (0.5–4.0 g/L) were altered to 25 °C and a pH of 12 in order to ascertain the impacts of SDBT dosage on equilibrium adsorption capacity (*q*_e_). The steady-state quantity of MB dye adsorbed (*q*_e_) for the same starting MB dye concentration rises as SDBT doses are lowered, as shown in Fig. 10. The equilibrium adsorption capacities (*q*_e_) in the removal of MB dye were calculated using SDBT adsorbents at various dosages (0.5–4.0 g/L), as shown in Fig. 10. These results range from 4.98 to 38.26, 9.97 to 62.30, 14.91 to 83.14, 19.85 to 90.70, 24.81 to 104.75, and 29.80 to 120.60 mg/g, respectively, for beginning MB dye concentrations (20, 40, 60, 80, 100, and 120 mg/L). As shown in Fig. 10, solutions with greater initial MB dye concentrations have a higher equilibrium adsorption capacity (*q*_e_) of MB dye on SDBT. As the adsorbent dosage rose, it was seen to decline. The initial concentration of the MB dyes had an impact on how efficiently they were absorbed from their aqueous solution, as seen in Fig. 10. In their research on the elimination of the dye Acid Yellow 11, El Nemr et al.^80^ noticed a similar pattern in their findings. The boundary layer effect is the first thing that happens to the MB dye molecules as they adhere to the SDBT surface. They diffuse from the boundary layer film to the surface, eventually mixing due to the adsorbent's porous composition.

#### SDBT dosage impact on MB dye adsorption

**Figure 11:**
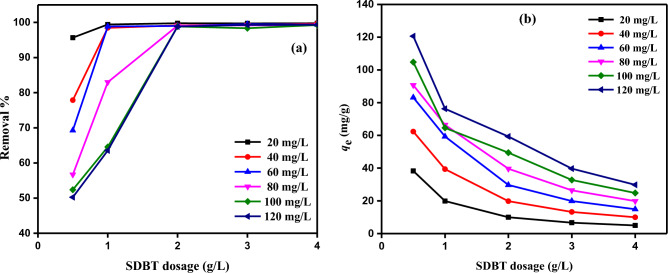
The SDBT different doses (0.5–4.0 g/L) impact using different starting MB dye concentrations (20–120 mg/L) (**a**) on the removal %; and (**b**) on the *q*_e_ (mg/g) at Temperature = 25 °C.

20–120 mg/L of MB concentration, 0.5–4.0 g/L of SDBT doses, 25 °C solution temperature, and 180 min. of adsorption duration at pH 12 solutions were used to test the impact of the adsorbent dosage on the removal of MB dye. Experimental findings are displayed in Fig. 11. According to experimental findings, increasing the dose of SDBT causes a rise in the MB dye removal percentage (%) (Fig. 11a) and a steady drop in the equilibrium (*q*_*e*_) values (Fig. 11b). Rapid filling of the active sites on the surface of the SDBT in the presence of highly concentrated dye molecules accounts for the release when the adsorbent dose is 2.0 g/L, and the initial MB dye concentration is 100–120 mg/L. As a result, 95–100% of the MB dye was removed (Fig. 11a). By increasing SDBT concentration from 0.5 to 4.0 g/L, the amount of MB dye adsorbed in equilibrium (*q*_e_) is calculated as 38.26 to 4.99, 62.30 to 9.97, 83.14 to 14.91, 90.70 to 19.85, 104.75 to 24.81, and 120.60 to 29.80 mg/g, respectively (Fig. 11b). The lowest amount of adsorption in equilibrium and the highest removal % of MB dye was 4.0 g/L of SDBT dosage.

### Adsorption isotherms

Equilibrium time and absorbate concentration (*C*_0_ in mg/L) are correlated with the absorbent mass at equilibrium (*q*_e_ in mg/g) in adsorption isotherms and are used to describe how absorbate molecules dispersed throughout solid–liquid phases^15,81^. To determine the optimal quantity of adsorbent to be used in experimental studies, the molecular fraction of the absorbate distributed in equilibrium (*q*_e_) between solid–liquid phases and isothermal data are used. This study utilized the LIM, FIM, and TIM isotherm models to examine the interactions between the SDBT and the MB dye^80^.

**Figure 12:**
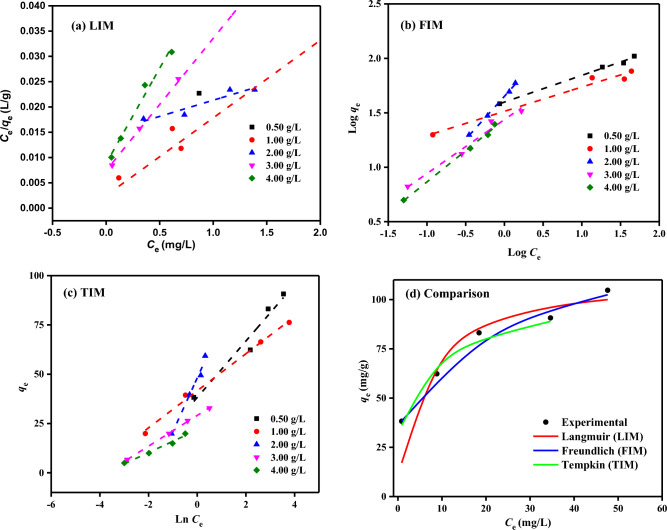
(**a**) LIM (**b**) FIM (**c**) TIM profiles for *C*_0_ of MB dye (20–120 mg/L) on SDBT doses (0.5–4.0 g/L) at 25 °C, (**d**) Rapprochement of measured and modelled isotherm of MB dye (*C*_0_: 20–120 mg/L, SDBT mass: 0.5 g/L, Temperature: 25 °C.

The absorption zones (*K*_L_) of the LIM constants and the computed adsorption capacity (*Q*_m_) for the absorption of MB dye by SDBT adsorbent are provided in Table 3. When the MB dye was absorbed onto the SDBT, a very high correlation coefficient (*R*^2^ = 0.900–1.000) was achieved in the LIM linear form, and the maximal single-layer capacity (*Q*_m_) was determined to be 156.25 mg/g. The intersection point of the *C*_e_/*q*_e_ vs. *C*_e_ graph (Fig. 12a) yields the LIM value of 1/*Q*_m_*K*_L_, and the slope is 1/*Q*_m_. The MB dye absorption on SDBT is demonstrated by the equilibrium adsorption constants (*K*_L_), which range between 0.43 and 6.16 L/mg and exhibit good correlation coefficients (*R*^2^ = 0.900–1.000). These findings demonstrate that MB dye molecules are absorbed on the SDBT surface in a single layer.

**Table 3:** Results of IM studied MB dye removal by SDBT adsorbent (MB (20–120 mg/L), SDBT mass (0.5–4.0 g/L) at 25 ± 2 °C).

IM	Parameters	SDBT adsorbent doses (g/L)				
		0.50	1.00	2.00	3.00	4.00
LIM	*Q*_*m*_ (mg/g)	109.89	64.93	156.25	38.16	26.59
	*K*_*L*_ × 10^3^	2.12	6.16	0.43	3.54	4.27
	*R*^2^	0.981	1.000	0.900	1.000	0.982
FIM	*1/n*	0.246	0.228	0.789	0.495	0.574
	*Qm* (mg/g)	87.48	66.87	568.16	133.69	174.01
	*K*_*F*_ (mg^1–1/n^ L^1/n^ g^–1^)	39.692	32.644	44.823	27.391	27.479
	*R*^2^	0.995	0.975	0.998	0.974	0.995
TIM	*A*_T_	14.45	83.78	5.84	41.99	44.26
	*B*_T_	14.31	9.39	27.26	7.78	5.73
	*R*^2^	0.959	0.996	0.986	0.999	0.982

Another model used to remove MB dye by SDBT adsorbent is the FIM. FIM assumes adsorption to the surface of the pollution is a heterogeneous phenomenon. The results of FIM model are shown in Table 3. The FIM's 1/*n*_F_ and log *K*_F_ values are given by the slope and intersection point of the log(*q*_e_)–log(*C*_e_) graph shown in Fig. 12b, respectively. *K*_F_ (L/g), one of the FIM constants known as the adsorption coefficient, indicates how much MB dye has been adsorbed on the adsorbent, given the unit equilibrium concentration. A higher *K*_F_ value indicates that the adsorbent has a greater capacity for adsorption. Additionally, the 1/*n* number must be smaller than 1.0 for the adsorbent to extract MB dye effectively. If the value of 1/*n* is less than 1.0, the SDBT adsorbent may physically remove the MB dye. As demonstrated by Table 3, all 1/*n* values are less than 1.0 and MB dye was easily adsorbed to SDBT adsorbent. Using Fig. 12b, the FIM isotherm correlation coefficients were calculated from the change in log(*q*_e_) as a function of log(*C*_e_). The *Q*_m_ of MB dye by SDBT adsorbent was determined as 568.16 mg/g in adsorbent with 2.0 g/L concentration, as seen in Table 3. FIM correlation coefficients (*R*^2^ = 0.974–0.998) were found to be relatively higher than LIM correlation coefficients (*R*^2^ = 0.900–1.000).

In this study, Temkin Model (TIM) explains how indirect adsorbent/adsorbate interactions affect adsorption and deals with heat exchange occurring during adsorbate adsorbed on the adsorbent surface, is also included. It is possible to calculate the parameters of TIM model (*A*_T_ and *B*_T_) with the help of the direct correlation between *q*_e_ and ln*C*_e_ shown in Fig. 12c. The slope of this graph gives *A*_T_ (g/L), and the intersection gives *B*_T_. Table 3 summarizes the TIM constants. The TIM correlation coefficients (*R*^2^ = 0.959–0.999) obtained to analyze the influence of temperature change on the MB dye removal using 0.5 g/L SDBT dose were quite high and showed that this model was suitable. The heat of adsorption (*B*_T_), which is released due to the absorbent-absorbate interaction, is of great importance in the MB dye adsorbing by SDBT adsorbent. Rapprochement of measured and modelled isotherm of MB dye was presented in Fig. 12d. When the obtained data were examined, the fact that the absorption heats were at very low levels showed that the adsorption was realized by physisorption.

### Best-fit IM via error function examination

The best model for the absorption of MB dye on SDBT adsorbent was chosen by comparing *R*^2^ of LIM, FIM, and TIM isotherm models with experimental data at equilibrium. Another method to choose the best-fit model is to compare the error functions. Marquardt's percent standard deviation (MPSD), the Chi-square error (X2), the average percent errors (APE), the sum of absolute errors (EABS), the root mean square errors (RMS), and the hybrid error function (HYBRID) can all be cited as examples^68,82^. When all error functions are compared, the TIM fits the experimental data quite well since the models with the closest values to zero will be the most appropriate model (Table 4).

**Table 4:** Some error function results of IMs used in the MB dye adsorption by SDBT.

IM	APE (%)	X^2^	Hybrid	MPSD	EABS	RMS
LIM	0.022	0.028	0.121	0.114	5.087	0.109
FIM	0.004	0.001	0.004	0.023	0.915	0.022
TIM	0.000	0.000	0.000	0.000	0.004	0.000

### Adsorption kinetic studies

**Figure 13:**
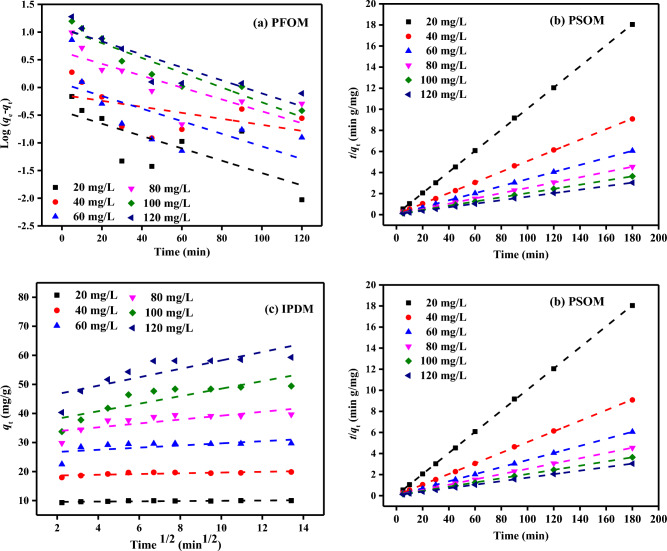
(**a**) PFOM (**b**) PSOM (**c**) IPDM (**d**) FDM of adsorption of MB dye by SDBT adsorbent (*C*_0_ = (20–120 mg/L), SDBT mass (2.0 g/L) at 25 ± 2 °C).

Time is an essential factor in the adsorbent's absorption efficiency (mg/g) at each dye concentration^83^. In this research, the adsorption equilibrium reached different times for various concentrations of MB dye. In addition, the starting dye concentration and the contact time also affected the absorption process at equilibrium^39,68,84^. To examine the absorption kinetics of MB dye on the SDBT surface, the obtained experimental data were tested to learn the control mechanism with pseudo-first-order (PFOM), pseudo-second-order (PSOM), film diffusion (FDM) and intraparticle diffusion (IPDM) kinetic models. Figure 13 shows adsorption kinetic plots for MB dye. In addition, Tables 5 and 6 summarize the experimental results, *R*^2^, and measured adsorption rate constants. The closer *R*^2^ values, ranging from zero (0) to one (1), are to one (1), which means that the model is more applicable to the experimental results. The fact that the *R*^2^ values are well below 0.900 and the values diverge many shows that the calculated *q*_e_ values are inappropriate with the experimental *q*_e_ values. Given the numbers in Table 5, it follows that the PFOM kinetic equation is inappropriate for the absorption of MB dye on SDBT. The rate constant, *k*_1_, and equilibrium adsorption capacity (*q*_e_), as calculated from the plot of Log(*q*_e_–*q*_t_) versus time (*t*) (Fig. 13a).

**Table 5:** PFOM and PSOM results of absorption of MB dye by SDBT (*C*_0_ = (20–120 mg/L), SDBT mass (0.5–4.0 g/L) at 25 ± 2 °C).

SDBT (g/L)	Parameter		PFOM			PSOM		
	MB dye (mg/L)	*q*_e_ (exp.)	*q*_e_ (calc.)	*k*_1_ × 10^3^	R^2^	*q*_*e*_ (calc.)	*k*_2_ × 10^3^	R^2^
0.50	20	38.26	10.14	0.01	0.655	37.74	8.08	0.992
	40	62.30	12.65	0.01	0.441	60.98	6.88	0.991
	60	83.14	25.23	0.01	0.340	81.30	3.27	0.997
	80	90.70	79.87	0.04	0.889	89.29	2.35	0.970
	100	104.75	50.52	2.15	0.931	101.01	0.97	0.990
	120	120.60	–	–	–	125.00	0.63	0.993
1.00	20	19.88	1.01	0.02	0.405	19.88	29.11	1.000
	40	39.88	8.91	0.01	0.768	39.53	16.37	0.999
	60	59.30	22.21	0.01	0.952	60.24	5.96	0.996
	80	66.39	18.83	0.01	0.602	68.49	4.00	0.979
	100	64.56	7.75	0.00	0.005	62.89	2.50	0.988
	120	76.25	76.52	0.18	1.000	79.37	1.57	0.992
2.00	20	9.97	2.70	0.03	0.545	9.98	115.54	1.000
	40	19.82	1.36	0.01	0.267	19.80	65.22	1.000
	60	29.70	1.18	0.03	0.479	29.76	24.44	1.000
	80	39.63	4.37	0.02	0.614	39.84	11.82	1.000
	100	49.42	11.69	0.03	0.892	50.25	3.91	1.000
	120	59.31	11.61	0.03	0.789	60.24	2.72	1.000
3.00	20	6.65	5.54	0.03	0.599	6.65	259.96	1.000
	40	13.24	2.87	0.02	0.376	13.25	145.79	1.000
	60	19.90	1.96	0.02	0.408	19.92	54.55	1.000
	80	26.44	2.23	0.03	0.650	26.60	26.52	1.000
	100	32.78	10.19	0.07	0.966	33.11	9.01	1.000
	120	39.71	2.27	0.02	0.435	39.84	6.22	1.000
4.00	20	4.99	8.40	0.03	0.577	4.99	461.68	1.000
	40	9.97	4.04	0.01	0.076	9.88	261.93	1.000
	60	14.91	3.70	0.01	0.011	12.02	149.83	1.000
	80	19.85	1.52	0.00	0.554	19.88	47.47	1.000
	100	24.81	1.28	0.04	0.829	24.88	15.97	1.000
	120	29.80	1.56	0.03	0.724	29.94	11.02	1.000

**Table 6:** IPDM and FDM results of absorption of MB dye by SDBT [*C*_0_ = (20–120 mg/L), SDBT mass (0.5–4.0 g/L) at 25 ± 2 °C].

Parameter		IPDM			FDM	
SDBT (g/L)	MB dye (mg/L)	*К*_*dif*_	*C*	R^2^	*К*_*FD*_	R^2^
0.50	20	0.972	24.82	0.821	0.025	0.763
	40	1.862	44.69	0.910	0.025	0.699
	60	2.621	49.55	0.902	0.024	0.738
	80	1.251	74.24	0.152	0.047	0.950
	100	1.727	78.35	0.846	0.025	0.935
	120	1.527	113.84	0.254	0.206	–
1.00	20	0.157	18.13	0.477	0.060	0.748
	40	1.037	26.17	0.953	0.034	0.816
	60	2.092	32.40	0.988	0.027	0.977
	80	1.992	39.30	0.911	0.026	0.732
	100	0.452	53.65	0.157	0.026	0.575
	120	1.177	74.41	0.730	0.200	0.999
2.00	20	0.047	9.45	0.582	0.068	0.773
	40	0.127	18.38	0.566	0.055	0.697
	60	0.370	26.00	0.345	0.068	0.768
	80	0.669	32.54	0.590	0.053	0.810
	100	1.295	35.58	0.716	0.049	0.902
	120	1.448	43.77	0.695	0.047	0.866
3.00	20	0.022	6.41	0.607	0.781	0.789
	40	0.068	12.50	0.467	0.062	0.714
	60	0.112	18.71	0.389	0.064	0.721
	80	0.412	22.21	0.506	0.059	0.813
	100	0.611	26.69	0.529	0.057	0.863
	120	0.596	33.65	0.395	0.660	0.762
4.00	20	0.015	4.82	0.615	0.075	0.769
	40	0.012	9.62	0.022	0.052	0.631
	60	− 0.165	14.65	0.030	0.057	0.545
	80	0.138	18.43	0.525	0.070	0.774
	100	0.190	22.91	0.430	0.081	0.859
	120	0.280	26.95	0.498	0.071	0.822

The MB dye amount adsorbed at equilibrium (*q*_e_) and its PSOM kinetic constant *k*_2_ were calculated by plotting the *t*/*q*_e_-time graph as seen in Fig. 13b. The *R*^2^ values for the MB dye and the linearity of the graphs as shown in Fig. 13b indicate that the adsorption mechanism fits the PSOM kinetics very well. In addition, when the *R*^2^ values of PSOM in Table 5 are investigated, it is seen that all of them are very close to unity.

Additionally, it was looked at how the adsorbate was transported from the MB dye solution to the SDBT surface using the intraparticle diffusion pattern^68,85^. As maintained by Weber and Morris^68,85^, the intraparticle diffusion step governs the absorption when the dashed lines displayed in the plot of *q*_t_ and root time (*t*) in Fig. 13c pass through the origin. Additionally, based on this graph, it is considered that film diffusion regulates the pace of the adsorption process if the dotted lines do not intersect the origin. The IPDM is used to explain solid–liquid adsorption solute transfer. The deposition of the adsorbate on the adsorbent takes place in three steps. The ions in the solution are first transported to the adsorbent surface through the liquid film if we were to list them. These molecules and ions on the adsorbent surface then moved and dispersed within the adsorbent. In the last phase, chemical processes occur in the adsorbent's active groups. The rate of absorption is measured by the slowest of these three processes because they all occur at various rates. Low *R*^2^ values are also present in IPDM and FDM. The intersection points and slope of the *q*_t_ versus *t*^0.5^ plot plotted in Fig. 13c give the *C* and *K*_*dif*_ values, respectively. None of the lines are straight, and all cross the origin due to the high C values. In Fig. 13d, it is shown that the film diffusion rises progressively as the volume of pores and SA of the SDBT diminish throughout the absorption process.

### Adsorption mechanism of MB dye ions by SDBT

**Figure 14:**
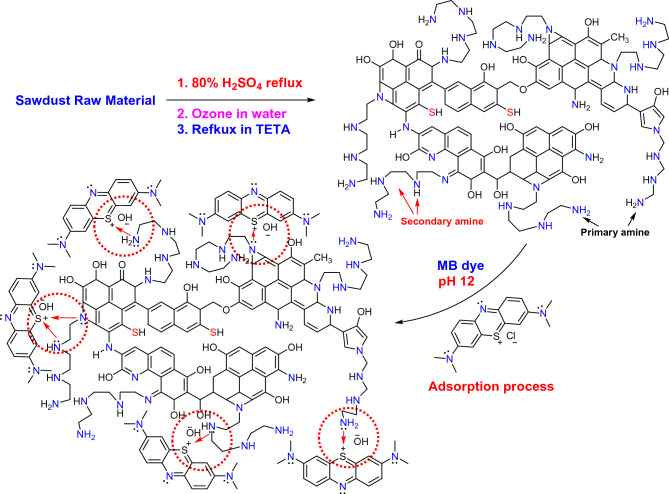
Probable mechanism for the MB dye adsorption onto the SDBT biochar.

The probable mechanism for the absorption of the MB dye ions by SDBT was explained in Fig. 14. After the dehydration of sawdust raw material with 80% H_2_SO_4_ and ozone oxidation followed by treatment with TETA at reflux, and many functional groups were formed on the adsorbent (SDBT) surface like C=N, amide N–H, hydroxyl O–H, C–N and SH groups as found from FTIR analysis. The MB dye ions absorption mechanism in a base medium (pH 12) may be achieved via physical interaction due to electrostatic interaction between the nitrogen lone pair on the SDBT surface and the positive charge on the sulfur atom of the MB dye, and then surface charge became positive which attract the hydroxyl ions from the base medium due to the basic pH 12.

In an alkaline environment, biochar's surface develops a negative charge that attracts positively charged dye molecules^2,3^. In addition, the OH^–^ ions in the solution interact with the carboxylic and phenolic functional groups on the surface of the biochar to produce negatively charged sites that can further adsorb positively charged dye molecules. Furthermore, a basic pH increases the solubility of dye molecules, facilitating their easier diffusion through the pores of the biochar and binding to the adsorption sites. Since the basic pH is crucial for promoting the adsorption of dye molecules onto biochar, biochar is a great approach to remove colour from industrial wastewater. The most important mechanism is the adsorption of ionizable organic molecules to the positively charged surface of the biochar through electrostatic contact^3^. How successfully an aqueous solution attracts or repels pollutants depends on its pH and ionic strength^3,15^.

Additionally, the pH of the solution has an impact on the ability of organic pollutants in industrial effluent to adsorb^17^. Parshetti et al.^19^ looked at the adsorption of textile colours in wastewater using biochar made from food waste. They discovered that the adsorption of dyes was improved by an alkaline pH. It was explained by the strong interaction between the positively charged dyes and the negatively charged sites on the surface of the biochar^19^. At pH 3, however, it was less effective at adsorbing organic dye because there was an excess of H^+^ that competed with the positive charges of the dye^19^. Both Tsai and Chen^21^ and Xu et al.^18^ made similar observations about how pH affects biochar's ability to adsorb substances. Thus, by changing the charged sites, the pH of the solution alters the ability of organic and inorganic contaminants from industrial wastewater to adsorb on biochar.

### Comparison with literature-based findings

In the literature review, different adsorbents' efficacy in removing textile dyes was compared to that of the SDBT adsorbent. Table 7 compares the *Q*_m_ (mg/g) of the SDBT utilised in this investigation and the maximum removal (%) of dye with previous findings mentioned in the literature. It is clear from Table 7 that the SDBT effectively absorbed the MB dye. Compared to the other adsorbents listed in Table 7, the SDBT's *Q*_m_ for the MB dye adsorption was a high 568.16 mg/g. This outcome could make the modified SDBT useful for treating wastewater containing dye.

**Table 7:** A comparison of the highest textile dye removal capabilities of some adsorbent materials.

Name of adsorbent	Textile dye	*Q*_m_ (mg/g)	Removal (%)	References
Mandarin-Biochar-TETA	Acid Yellow 11	384.62	96.76	^15^
Cellulose-based biocomposite film	Methylene Blue	146.81	–	^86^
Ethylenediamine-Modified Peanut Husk	Sunset Yellow	117.70	–	^87^
Sludge-Rice Husk Biochar	Direct Red	59.77 (DR)	–	^88^
	Acid Orange II	42.12 (AOII)	–	
	React Blue 19	38.46 (RB19)	–	
	Methylene Blue	22.59 (MB)	–	
Mandarin Nanoporous Carbon	Methylene Blue	313.00 (MB)	–	^89^
	Metanil Yellow	455.00 (MY)	–	
Activated spent tea	Methylene Blue	104.20 (MB)	–	^90^
Mandarin-Biochar-O_3_-TETA	Acid Red 35	476.19 (AR35)	97.5	^17^
N-doped Biochars derived from Phragmites Australis	Acid Red 18	134.17 (AR18)	–	^91^
Carbonized Mandarin Peel	Methylene Blue	196.08 (MB)	99.77 (MB)	^92^
	Methyl Orange	–	79.87 (MO)	
Pea Peels-AC	Acid Yellow 11	515.46 (AY11)	99.10	^80^
Jute stick derived activated carbon	Methylene Blue	384.60 (MB)	–	^93^
Mandarin Shells	Basic Blue 9	294.00 (BB9)	–	^60^
	Acid Yellow 36	417.00 (AY36)	–	
Amphoteric modified bentonite	Acid Yellow 11	50.25 (AY11)	–	^94^
Juncus effusus (JE)-based adsorbent	Acid Yellow 11	526.30 (AY11)	93.44 (AY)	^95^
	Reactive Red 195	452.50 (RR195)	99.23 (RR)	
	Direct Blue 15	255.10 (DB15)	95.60 (DB)	
Macore Fruit Shells	Methyl Orange	3.42 (MO)	82.73 (MO)	^57^
	Methylene Blue	10.61 (MB)	91.31 (MB)	
Biochar from gasification of Wood Wastes	Indosol Black NF1200	185.00	99.00	^96^
Mandarin Peel Biochar	Methyl Orange	16.27 (MO)	99.00 (MO)	^97^
	Fast Green	12.44 (FG)	99.00 (FG)	
Sawdust carbon	Cu	–	79%	^98^
Mandarin-CO-TETA	Acid Brown 14	416.67	86.9%	^99^
SDBT	Methylene Blue	568.16 (MB)	100.00	This work

### Regeneration of SDBT

**Figure 15:**
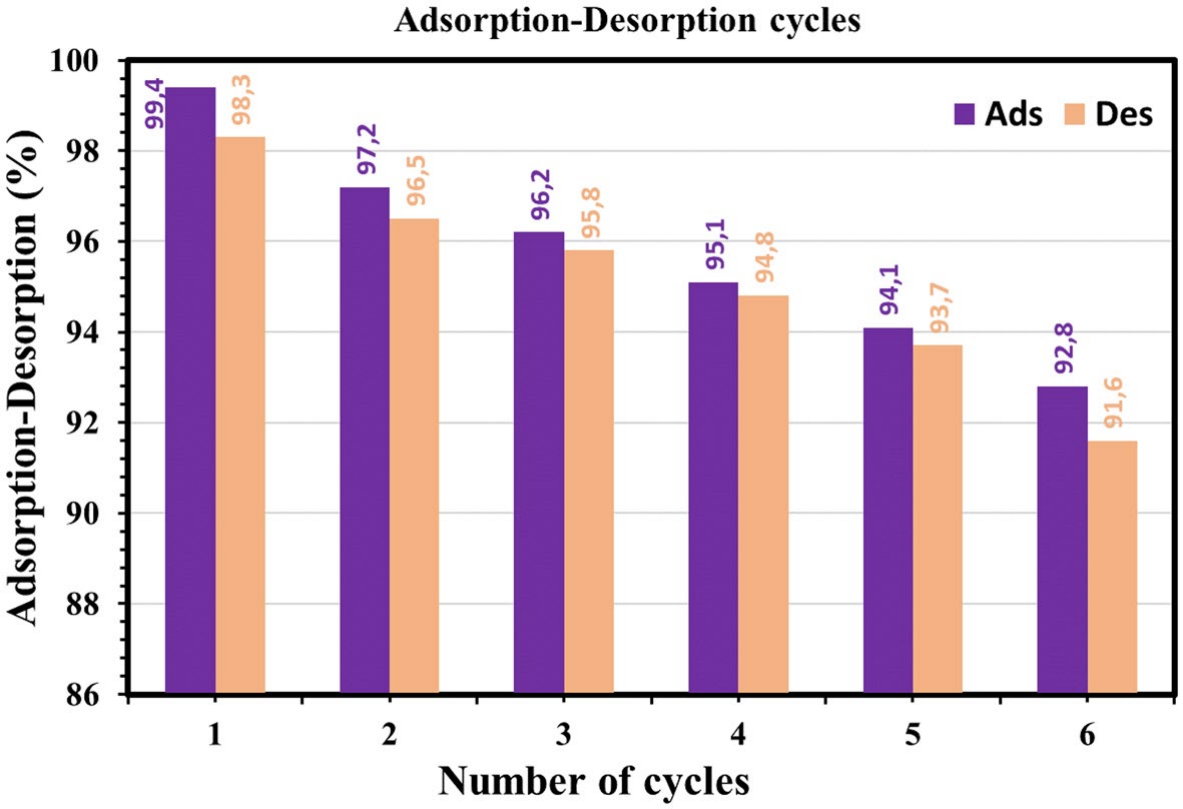
MB dye was desorption % from SDBT by 0.1 M HCl and NaOH, and SDBT regenerated was used to promote MB dye absorption cycles for six times (20 mg/L MB, 1.0 g/L SDBT).

Desorption tests are very important to test the viability and reusability of the produced adsorbents. To test this, desorption tests of MB dye from SDBT in an elution medium with 0.1M HCl and NaOH were performed. The proportion of desorption reduced as the number of regeneration cycles increased, as seen in Fig. 15. There were six consecutive adsorption/desorption cycles using regenerated SDBT. The amount of adsorption did not vary much throughout the cycles, but it dropped by 6.64% after six generations. This finding suggests that SDBT is an effective long-term MB dye removal adsorbent.

## Conclusion

It has been demonstrated in this work that sawdust, a waste product of woodworking operations, can be used to create an affordable and efficient adsorbent material. Dry sawdust biochar was prepared by heating with 80% sulfuric acid at reflux, followed by oxidation with ozone and subsequent treatment with triethylenetetramine (TETA) at reflux, which is ready to be tested for the removal of MB dye. The factors affecting the MB dye adsorption by the SDBT biochar. SDBT dose, contact time, pH and starting MB dye concentration were investigated, and it was determined that the optimum pH was 12. The maximum elimination of MB dye and the least amount of adsorption (*q*_e_) at equilibrium were determined by working with an SDBT dose of 2.0 g/L concentration. FIM and TIM models performed better than the LIM model in removing MB dye. The *Q*_m_ of SDBT adsorbent was calculated as 568.16 mg/g using the FIM. The removal of MB dye was accomplished through physisorption since there was little ionic contact between the adsorbent and adsorbate and the absorption temperature was low. As a result of this study's findings, it is possible to effectively and economically remove MB dye from wastewater using SDBT as an adsorbent.

## Data Availability

The datasets used in this investigation are accessible for review upon request from the paper's corresponding author.
